# Evaluating increasing levels of sodium diformate in diets for nursery and finishing pigs on growth performance, fecal dry matter, and carcass characteristics

**DOI:** 10.1093/tas/txae085

**Published:** 2024-05-18

**Authors:** Katelyn N Gaffield, Gracie J Becker, Jessica L Smallfield, Joel M DeRouchey, Mike D Tokach, Jason C Woodworth, Robert D Goodband, Troy Lohrmann, Christian Lückstädt, Mariana B Menegat, Mary Liebenstein, Matt Allerson, Jordan T Gebhardt

**Affiliations:** Department of Animal Sciences and Industry, College of Agriculture, Kansas State University, Manhattan, KS 66506-0201, USA; Department of Animal Sciences and Industry, College of Agriculture, Kansas State University, Manhattan, KS 66506-0201, USA; Department of Animal Sciences and Industry, College of Agriculture, Kansas State University, Manhattan, KS 66506-0201, USA; Department of Animal Sciences and Industry, College of Agriculture, Kansas State University, Manhattan, KS 66506-0201, USA; Department of Animal Sciences and Industry, College of Agriculture, Kansas State University, Manhattan, KS 66506-0201, USA; Department of Animal Sciences and Industry, College of Agriculture, Kansas State University, Manhattan, KS 66506-0201, USA; Department of Animal Sciences and Industry, College of Agriculture, Kansas State University, Manhattan, KS 66506-0201, USA; Quality Technology International, Inc., Elgin, IL 60123, USA; ADDCON, 06749 Bitterfeld-Wolfen, Germany; Holden Farms, Inc., Northfield, MN 55057, USA; Holden Farms, Inc., Northfield, MN 55057, USA; Holden Farms, Inc., Northfield, MN 55057, USA; Department of Diagnostic Medicine/Pathobiology, College of Veterinary Medicine, Kansas State University, Manhattan, KS 66506-0201, USA

**Keywords:** acidifier, feed additive, finishing pig, nursery pig, organic acid, sodium diformate

## Abstract

Two studies were conducted to evaluate the effects of sodium diformate in swine diets. For Exp. 1, 360 barrows (DNA 200 × 400; initially 5.9 ± 0.06 kg) were used in a 38-d study. At weaning, pigs were randomly assigned to pens with five pigs per pen. Each pen was allocated to one of six treatments with 12 pens per treatment. Treatments were formulated to provide none, 0.40%, 0.60%, 0.80%, 1.00%, or 1.20% sodium diformate added at the expense of corn. Diets were fed in three phases: phase 1 from weaning to day 9, phase 2 from days 9 to 24, and phase 3 from days 24 to 38. From days 0 to 24 (phases 1 and 2), increasing sodium diformate increased (linear, *P* = 0.001) gain-to-feed (**G:F**). However, sodium diformate did not affect average daily gain (**ADG**) or average daily feed intake (**ADFI**). From days 24 to 38 (phase 3) and overall (days 0 to 38), there was no evidence of differences due to increasing sodium diformate for any growth response criteria. There was no evidence for differences in fecal dry matter (**DM**) on day 9. However, fecal DM decreased (linear, *P* < 0.05; quadratic, *P* = 0.097) as sodium diformate increased on day 24. In Exp. 2, 2,200 pigs (Duroc sire [PIC 800 or DNA 600] × PIC Camborough; initially 24.2 ± 0.30 kg) were used in a 117-d growth trial. Pens of pigs (25 pigs per pen) were randomly assigned to one of four treatments with 22 pens per treatment. Treatments were formulated with additions of none, 0.25%, 0.50%, or 0.75% sodium diformate. Diets were fed in six phases from 24 to 141 kg. For period 1 (days 0 to 32), ADFI tended to decrease then increase (quadratic, *P* = 0.081) with increasing sodium diformate, whereas G:F increased then decreased (quadratic, *P* < 0.001) with increasing sodium diformate. For period 2 (days 32 to 60), there was no evidence for differences in ADG or ADFI; however, there was a tendency for G:F to increase then decrease (quadratic, *P* = 0.093) with increasing sodium diformate. From days 60 to 93, increasing sodium diformate increased (linear, *P* < 0.01) ADG and ADFI. From days 93 to 117, increasing sodium diformate increased (linear, *P* < 0.05) ADG, ADFI, and G:F. Overall (days 0 to 117), pigs fed increasing sodium diformate had increased (linear, *P* < 0.01) ADG and a tendency for increased (linear, *P* = 0.075) ADFI; however, there was no evidence for differences in G:F. There were no treatment differences for any carcass characteristic. In summary, increasing sodium diformate may increase G:F in the early nursery and improve ADG after day 60 (approximately 82 kg) in the finishing period.

## Teaser

Increasing sodium diformate improved feed efficiency in the early nursery period and improved average daily gain in the late finishing period.

## INTRODUCTION

Organic acids, such as formic acid, are commonly used as acidifiers in swine diets. Dietary acidifiers have the potential to lower gastrointestinal tract pH, which in turn can improve nutrient digestion, growth performance, and alter the gut microbiota of pigs (Suiryanrayna and Ramana, 2015). Within dietary acidifiers, formic acid has one of the highest acidification potentials (Lawlor et al., 2005). This high acidification potential indicates formic acid may improve both swine health and growth performance. However, due to the handling characteristics of pure formic acid, it is commonly fed in the form of calcium, sodium, or potassium salts. Sodium diformate is a relatively new salt of formic acid being introduced into the U.S. market and is the result of combining sodium formate and formic acid into a single, free-flowing product.

The use of formic acid in the form of potassium salts has been extensively studied in both nursery (Tung and Pettigrew, 2006; Poeikhampha and Bunchasak, 2011; Htoo and Molares, 2012) and finishing pigs (Overland, et al., 2000; Mroz et al., 2002). More specifically, potassium diformate has been shown to consistently increase average daily gain (**ADG**) and gain-to-feed ratio (**G:F**; Poeikhampha and Bunchasak, 2011; Htoo and Molares, 2012), benefit intestinal morphology (Poeikhampha and Bunchasak, 2011), and exhibit antimicrobial properties (Canibe et al., 2001) when fed to nursery pigs. Potassium diformate has also been shown to improve both growth performance and carcass characteristics when fed to finishing pigs (Overland et al., 2000). Despite the positive results reported when utilizing potassium diformate in swine diets, there is currently limited research evaluating the use of other formic acid salts, such as sodium diformate.

There are a wide variety of acidifiers on the market, each with different compositions and acidification properties. Therefore, it is important to validate the effects of each new acidifier on pig performance before implementation into commercial diets. Due to the positive effects reported with other formic acid salts, sodium diformate has the potential to act as another effective acidifier in swine diets. However, there is currently limited information evaluating the use of sodium diformate in swine diets. Recently, a study reported improvements in growth performance when sodium diformate was included at 1.2% in nursery diets (Hutchens et al., 2021). Therefore, further research is needed to validate these nursery results as well as provide data on the effects of sodium diformate in finishing pig diets.

## MATERIALS AND METHODS

The Kansas State University Institutional Animal Care and Use Committee approved the protocols used in these experiments.

### Experiment 1

The study was conducted at the Kansas State University Segregated Early Weaning Facility located in Manhattan, KS where pigs were housed in two identical, environmentally controlled barns. Pens (1.2 × 1.2 m) had metal tri-bar floors and housed five pigs per pen allowing approximately 0.30 m^2^/pig. Each pen contained a cup waterer and a four-hole, dry self-feeder which provided ad libitum access to feed and water.

A total of 360 weanling barrows (DNA 200 × 400; initially 5.9 ± 0.06 kg) were used in a 38-d study. Pigs were randomly assigned to pens and pens were allotted to one of six dietary treatments with 12 pens per treatment. Diets were fed in three phases: phase 1 from weaning to day 9, phase 2 from days 9 to 24, and phase 3 from days 24 to 38. Dietary treatments were formulated to provide none, 0.40%, 0.60%, 0.80%, 1.00%, and 1.20% sodium diformate (Formi NDF, ADDCON Nordic AS, Porsgrunn, Norway) added at the expense of corn (Table 1). For phases 1 and 2, single-base diets were manufactured at the Kansas State University O.H. Kruse Feed Technology Innovation Center, Manhattan, KS. The base diet was then used to manufacture the six treatment diets through additions of sodium diformate at the expense of corn. For phase 3, complete diets were manufactured with a total of two batches per treatment. All diets were fed in meal form.

**Table 1.** Diet composition (as-fed basis), Exp. 1^1^

Item, %	Phase 1	Phase 2	Phase 3
Ingredients, %			
Corn	44.85	50.12	64.18
Soybean meal	17.41	23.76	31.78
Spray-dried whey	10.00	—	—
Whey permeate	10.00	10.00	—
Dried distillers grains with solubles	5.00	7.50	—
Fish meal	2.50	—	—
Fermented soybean meal^2^	4.00	3.85	—
Spray-dried bovine plasma	2.00	—	—
Soybean oil	1.00	1.00	—
Monocalcium P	0.80	1.00	1.00
Calcium carbonate	0.41	0.40	0.88
Zinc oxide	0.40	0.25	—
Sodium chloride	0.30	0.50	0.60
Vitamin premix^3^	0.25	0.25	0.25
Trace mineral premix^4^	0.15	0.15	0.15
Phytase^5^	0.08	0.08	0.08
l-Lys-HCL	0.40	0.55	0.50
dl-Met	0.19	0.23	0.21
l-Thr	0.17	0.22	0.21
l-Trp	0.02	0.04	0.04
l-Val	0.08	0.12	0.13
Sodium diformate^6^	+/−	+/−	+/−
Total	100	100	100
Calculated analysis^7^			
Standardized ileal digestible (SID) amino acids, %			
Lys, %	1.35	1.35	1.35
Ile:Lys	57	57	56
Leu:Lys	120	118	115
Met:Lys	36	38	36
Met and Cys:Lys	56	56	58
Thr:Lys	64	63	63
Trp:Lys	19.2	19.2	19.3
Val:Lys	70	70	70
Total Lys, %	1.51	1.51	1.50
NE, kcal/kg	2,544	2,504	2,418
SID Lys:NE, g/Mcal	5.30	5.39	5.58
Crude protein, %	21.5	21.5	21.3
Ca, %	0.66	0.54	0.71
P, %	0.66	0.61	0.61
STTD P, %	0.59	0.51	0.48

^1^Phase 1 diets were fed from 5.9 to 7.1 kg, phase 2 diets were fed from 7.1 to 13.4 kg, and phase 3 diets were fed from 13.4 to 21.7 kg.

^2^MEPro, Prairie Aquatech, Brookings, SD.

^3^Provided per kilogram of diet: 4,134 IU vitamin A, 1,653 IU vitamin D, 44 IU vitamin E, 3 mg vitamin K, 0.03 mg vitamin B12, 50 mg niacin, 28 mg pantothenic acid, 8 mg riboflavin.

^4^Provided per kilogram of diet: 110 mg Zn from zinc sulfate, 110 mg Fe from iron sulfate, 33 mg Mn from manganese oxide, 17 mg Cu from copper sulfate, 0.30 mg I from calcium iodate, 0.30 mg Se from sodium selenite.

^5^Ronozyme HiPhos 2700 GT, DSM Nutritional Products, Parsippany, NJ was included at 2,000 FTU/kg to provide an estimated release of 0.12% STTD P for all diets.

^6^Sodium diformate (Formi NDF, ADDCON Nordic AS, Porsgrunn, Norway) was included at none, 0.40%, 0.60%, 0.80%, 1.00%, and 1.20% at the expense of corn.

^7^Nutrient values from the NRC (2012) were used for the calculated analysis.

NE, net energy; STTD P, standardized total tract digestible phosphorus.

Pig weights and feed disappearance were measured on days 0, 9, 18, 24, 31, and 38 to determine ADG, average daily feed intake (**ADFI**), and G:F. Feces were collected on days 9 and 24 from three pigs per pen via rectal massage to determine fecal dry matter (**DM**). Samples were stored at −20 °C until analyzed. Fecal samples were dried at 55 °C for 48 h and loss of weight was used to determine percentage DM.

### Experiment 2

Two rooms at a commercial research site located in south-central Minnesota (Holden Farms, Inc., Northfield, MN) were used for this experiment. Barns had completely slatted, concrete flooring and contained pens (3 × 5.5 m) equipped with a three-hole feeder (Thorp Equipment, Inc., Thorp, WI) and double-sided pan waterer. Pigs were provided ad libitum access to feed and water. A computerized feeding system (FeedPro; Feedlogic Corp., Willmar, MN) provided daily feed additions.

A total of 2,200 pigs (Duroc sire [PIC 800 or DNA 600] × PIC Camborough; initially 24.2 ± 0.30 kg) were used to conduct a 117-d growth trial. Pens of pigs (25 pigs per pen) were randomly assigned to one of four dietary treatments in a randomized complete block design with 22 pens per treatment. Dietary treatments were corn–soybean meal based with the addition of none, 0.25%, 0.50%, or 0.75% sodium diformate. All diets were manufactured at Bixby Feed Mill, Inc. (Blooming Prairie, MN) and fed in meal form. Diets were fed in six phases from 24 to 34, 34 to 66, 66 to 88, 88 to 111, 111 to 120, and 120 to 141 kg. Nutrients for all treatment diets were formulated to meet or exceed the NRC (2012) requirement estimates for growing and finishing pigs in each appropriate weight range (Table 2).

**Table 2.** Composition of experimental diets (as-fed basis), Exp. 2^1^

Item	Phase 1	Phase 2	Phase 3	Phase 4	Phase 5	Phase 6
Ingredients, %						
Corn	42.96	52.96	60.73	64.54	69.38	82.73
Soybean meal	24.50	14.50	6.75	3.00	3.25	15.50
Dried distillers grains with solubles	30.00	30.00	30.00	30.00	25.00	—
Monocalcium P	0.05	—	—	—	—	0.05
Calcium carbonate	1.20	1.10	1.10	1.10	1.10	0.80
Sodium chloride	0.50	0.50	0.50	0.50	0.50	0.50
l-Lys-HCl	0.36	0.49	0.51	0.49	0.45	0.19
Thr^2^	0.06	0.12	0.12	0.11	0.11	0.07
l-Trp	—	0.03	0.05	0.05	0.04	—
Vitamin premix with phytase^3^	0.20	0.15	0.13	0.13	0.10	0.10
Trace mineral premix	0.15	0.13	0.10	0.10	0.08	0.08
Copper sulfate	0.03	0.03	0.03	—	—	—
Sodium diformate^4^	+/−	+/−	+/−	+/−	+/−	+/−
Total	100	100	100	100	100	100
Calculated analysis^5^						
Standardized ileal digestible (SID) amino acids, %						
Lys, %	1.15	1.02	0.86	0.75	0.72	0.72
Ile:Lys	68	61	58	58	58	66
Leu:Lys	167	166	177	191	189	163
Met:Lys	30	29	31	33	33	30
Met and Cys:Lys	57	56	59	63	63	60
Thr:Lys	63	63	63	64	65	65
Trp:Lys	18	18	18	18	18	18
Val:Lys	78	73	72	75	74	76
His:Lys	46	43	43	44	44	47
Total Lys, %	1.34	1.18	0.99	0.88	0.83	0.79
NE, kcal/kg	2,465	2,489	2,504	2,513	2,533	2,604
SID Lys:NE, g/Mcal	4.67	4.11	3.42	3.00	2.83	2.76
Crude protein, %	23.2	19.5	16.4	15.0	14.0	13.2
Ca, %	0.64	0.58	0.53	0.51	0.49	0.43
STTD P, %	0.41	0.38	0.35	0.34	0.31	0.26

^1^Phases were fed from approximately 24 to 34, 34 to 66, 66 to 88, 88 to 111, 111 to 120, and 120 to 141 kg BW, respectively.

^2^Thr Pro; CJ America-Bio, Downers Grove, IL.

^3^Phytase was included at 880 FTU/kg in phase 1 (estimated 0.14% STTD P release), 660 FTU/kg in phase 2 (0.14% STTD P release), 550 FTU/kg in phases 3 and 4 (0.13% STTD P release), and 440 FTU/kg in phases 5 and 6 (0.12% STTD P release).

^4^Formi NDF (ADDCON Nordic AS, Porsgrunn, Norway) at none, 0.25%, 0.50%, and 0.75% of the diet was included at the expense of corn.

^5^Nutrient values from the NRC (2012) were used for all the calculated analysis.

NE, net energy; STTD P, standardized total tract digestible phosphorus.

Pens of pigs were weighed every 2 wk and feed disappearance was measured to determine ADG, ADFI, and G:F. Two weeks prior to the end of the experiment, the heaviest four pigs per pen were visually identified, weighed, and marketed. These pigs were used to determine growth performance but were not included in carcass collection. The remaining pigs were weighed and marketed at the completion of the study. Pigs were transported to a U.S. Department of Agriculture-inspected packing plant (Tyson Fresh Meats, Waterloo, IA). Hot carcass weight (**HCW**), loin depth, and backfat measurements were collected. Carcass yield was determined using the pen average HCW divided by the pen average final live weight. A proprietary equation from the packing plant was used to calculate the percentage lean.

For both studies, under the circumstance where a pig died or needed to be removed from the study due to the inability to overcome sickness or injury, the weight of the pig and feed consumption up until that date were recorded and used to adjust the growth performance calculations accordingly.

### Statistical Analysis

Data from both experiments were analyzed using the GLIMMIX procedure of SAS (v. 9.4, SAS Institute, Inc., Cary, NC). Data from Exp. 1 were analyzed as a completely randomized design with pen as the experimental unit, barn as a random effect, and treatment as the fixed effect. Data from Exp. 2 were analyzed as a randomized complete block design with pen as the experimental unit, treatment serving as the fixed effect, and initial body weight (**BW**) serving as a blocking factor. For both experiments, linear and quadratic contrasts were used to test for increasing levels of sodium diformate. Contrast statements were used to test for the main effects of treatment, day, and interaction between treatment and day on fecal DM. For carcass characteristics, individual carcasses served as the observational unit, pen served as the experimental unit, initial BW served as the blocking factor, HCW was included as a covariate, and pen was included as a random effect to account for subsampling. Additionally, linear and quadratic contrasts were used to analyze carcass characteristics including backfat, loin depth, and percentage lean. All results were considered significant with *P* ≤ 0.05 and marginally significant with *P* ≤ 0.10.

## Results

### Experiment 1

From days 0 to 24 (phases 1 and 2), increasing sodium diformate increased (linear, *P* = 0.001) G:F (Table 3). However, sodium diformate did not affect ADG or ADFI. From days 24 to 38 (phase 3) and the overall study, there was no evidence of differences for any of the growth performance criteria. Sodium diformate did not affect BW at any timepoint in the trial. Overall mortality was 0.8% and was not influenced by treatment (data not shown).

**Table 3.** Effects of increasing sodium diformate on nursery pig performance and fecal dry matter, Exp. 1^1^

Item	Sodium diformate, %^2^						SEM	P	
	0	0.40	0.60	0.80	1.00	1.20		Linear	Quadratic
BW, kg									
Day 0	5.9	5.9	5.9	5.9	5.9	5.9	0.06	0.905	0.911
Day 24	13.3	13.2	13.4	13.6	13.4	13.3	0.20	0.671	0.630
Day 38	21.7	21.6	21.8	21.8	21.6	21.7	0.33	0.980	0.956
Days 0 to 24 (phases 1 and 2)									
ADG, g	306	302	312	320	313	304	8.5	0.683	0.420
ADFI, g	393	386	396	396	391	374	9.2	0.356	0.237
G:F, g/kg	779	783	788	807	799	812	8.2	0.001	0.629
Days 24 to 38 (phase 3)									
ADG, g	600	599	602	583	585	597	15.8	0.511	0.803
ADFI, g	852	867	858	849	848	856	17.0	0.822	0.792
G:F, g/kg	705	692	702	686	689	697	9.7	0.350	0.421
Days 0 to 38 (overall)									
ADG, g	414	412	419	417	413	410	8.8	0.822	0.626
ADFI, g	561	563	566	563	560	548	11.0	0.443	0.332
G:F, g/kg	738	731	740	740	738	748	5.6	0.186	0.293
Fecal DM, %^3^									
Day 9	26.26	26.77	26.85	26.91	24.80	25.41	1.146	0.299	0.287
Day 24	24.21	25.70	25.32	23.80	21.92	23.41	0.914	0.028	0.097

^1^A total of 360 weanling barrows (DNA 200 × 400, DNA; initially 5.9 ± 0.06 kg) approximately 21 d of age were used in a 38-d experiment with five pigs per pen and 12 pens per treatment.

^2^Formi NDF, ADDCON Nordic AS, Porsgrunn, Norway.

^3^Feces from three piglets from each pen were pooled, weighed, and dried to measure fecal dry matter. Treatment × day, *P* = 0.749; Treatment, *P* = 0.120; Day, *P* < 0.001.

DM, dry matter.

There was no evidence of an interaction between treatment and day for fecal DM. There was no evidence for differences in fecal DM on day 9. However, fecal DM decreased (linear, *P* = 0.028; quadratic, *P* = 0.097) as sodium diformate increased on day 24. Additionally, there was evidence for a main effect of day (*P* < 0.001) with fecal DM being lower on day 24 compared to day 9.

### Experiment 2

For period 1 (days 0 to 32), ADFI tended to decrease then increase (quadratic, *P* = 0.081) with increasing sodium diformate, whereas G:F increased then decreased (quadratic, *P* < 0.001) with increasing sodium diformate (Table 4). For period 2 (days 32 to 60), there was no evidence for differences in ADG or ADFI; however, there was a tendency for G:F to increase then decrease (quadratic, *P* = 0.093) with increasing sodium diformate. From days 60 to 93, increasing sodium diformate increased (linear, *P* < 0.01) ADG and ADFI. Additionally, from days 93 to 117, increasing sodium diformate increased (linear, *P* < 0.05) ADG, ADFI, and G:F. Overall (days 0 to 117), pigs fed increasing sodium diformate had increased (linear, *P* < 0.01) ADG and a tendency for increased (linear, *P* = 0.075) ADFI; however, there was no evidence for differences in G:F. Furthermore, increasing sodium diformate increased (linear, *P* = 0.005) final BW on day 117.

**Table 4.** Effects of increasing sodium diformate on growth performance and carcass characteristics of finishing pigs, Exp. 2^1^

Item	Sodium diformate, %^2^				SEM	P	
	0	0.25	0.50	0.75		Linear	Quadratic
BW, kg							
Day 0	24.2	24.2	24.3	24.2	0.30	0.808	0.538
Day 32	53.4	53.5	53.2	53.3	0.40	0.445	0.834
Day 60	81.8	82.2	81.9	81.9	0.47	0.954	0.359
Day 93	116.3	117.1	117.0	117.1	0.48	0.118	0.338
Day 117	138.8	139.8	140.2	140.4	0.70	0.005	0.307
Period 1 (days 0 to 32)							
ADG, kg	0.91	0.91	0.90	0.90	0.005	0.287	0.641
ADFI, kg	1.72	1.70	1.70	1.71	0.014	0.424	0.081
G:F, g/kg	528	537	533	528	2.5	0.735	<0.001
Period 2 (days 32 to 60)							
ADG, kg	1.01	1.03	1.03	1.02	0.008	0.646	0.166
ADFI, kg	2.46	2.48	2.47	2.47	0.018	0.810	0.580
G:F, g/kg	412	415	416	413	1.9	0.625	0.093
Period 3 (days 60 to 93)							
ADG, kg	1.04	1.05	1.06	1.07	0.006	0.004	0.970
ADFI, kg	3.01	3.02	3.05	3.06	0.016	0.008	0.817
G:F, g/kg	348	347	348	349	2.0	0.462	0.797
Period 4 (days 93 to 117)							
ADG, kg	1.07	1.09	1.10	1.12	0.015	0.003	0.708
ADFI, kg	3.59	3.63	3.63	3.66	0.022	0.017	0.798
G:F, g/kg	299	301	304	305	3.5	0.035	0.782
Overall (days 0 to 117)							
ADG, kg	1.00	1.01	1.01	1.02	0.004	0.004	0.236
ADFI, kg	2.60	2.61	2.62	2.63	0.014	0.075	0.905
G:F g/kg	385	387	388	387	1.9	0.313	0.212
Carcass characteristics							
HCW, kg	101.7	102.4	103.1	102.4	0.60	0.168	0.107
Carcass yield, %	73.4	73.3	73.4	72.9	0.22	0.117	0.334
Backfat, mm^3^	13.2	13.1	12.9	13.0	0.15	0.207	0.380
Loin depth, mm^3^	73.0	73.2	72.7	72.5	0.46	0.220	0.545
Lean, %^3^	57.3	57.3	57.3	57.2	0.08	0.719	0.383
Removals, %	1.82	0.91	0.91	1.46	0.570	0.666	0.144
Mortality, %	1.09	0.91	1.09	1.46	0.511	0.545	0.562
Mortality and removals, %	2.91	1.82	2.00	2.91	0.717	0.934	0.133

^1^A total of 2,200 pigs (initially 24.2 ± 0.30 kg) were used in a 117-d growth trial with 25 pigs per pen and 22 replicates per treatment.

^2^Formi NDF, ADDCON Nordic AS, Porsgrunn, Norway.

^3^Adjusted using HCW as covariate.

HCW, hot carcass weight.

There was no evidence of differences observed for HCW, carcass yield, backfat, loin depth, or percentage lean due to increasing sodium diformate. Overall removals and mortalities were 1.3% and 1.1%, respectively, and were not influenced by treatment.

## DISCUSSION

As the swine industry continues to decrease the usage of antibiotic growth promoters and pharmacological levels of Zn, multiple feed additives including acidifiers, probiotics, prebiotics, and direct-fed microbials are being studied in order to help maintain pig health and performance (Overland, 2001; Liu et al., 2018). Of the various feed additives, acidifiers have been observed to elicit a positive response on growth performance (Kil et al., 2011; Lückstädt and Mellor, 2011; Rao et al., 2023). An extensive review by Kil et al. (2011) evaluating the use of acidifiers in weanling pig diets found that acidifiers elicit a positive response in growth performance when included during the nursery period; however, there is a high level of variation in response. Furthermore, a review by Rao et al. (2023) focused on the effects of different feed additives on finishing pig growth performance concluded that acidifiers had the potential to positively impact feed efficiency when compared to other feed additives. However, in the feed additive review, implementing acidifiers in finishing diets had a wide range in response with an impact on ADG ranging from −14.9% to 11.4% and an impact on feed efficiency ranging from −9.7% to 11.3% (Rao et al., 2023). For both reviews, many factors may be contributing to the variation in response including acidifier type, inclusion level, protein source, protein level, and health status. Therefore, current research is needed to focus on identifying acidifiers that elicit a positive response in swine diets and further understanding their use in different stages of production.

Formic acid is an acidifier characterized as a simple carboxylic acid. Formic acid has gained attention for use within the swine industry due to its high acidification potential (Lawlor et al., 2005). However, due to the corrosiveness and handling characteristics of formic acid, it is often fed in salt form. Forms of formic acid can include calcium, sodium, or potassium salts. There is currently limited research evaluating the use of multiple formic acid salts, including sodium diformate. The sodium diformate product used in this series of trials was a combination of 57% sodium formate and 38.5% formic acid.

Although acidifiers have shown to be variable in response, they are commonly thought to have the most consistent effects on growth performance when utilized during the nursery period. Weaning is a crucial period of a pig’s life where they are challenged with a multitude of factors including changes in environment, diet, and physiological development. Specifically, due to the substantial change in diet, newly weaned pigs exhibit reduced stomach acid secretions (Pluske, 2016). Addition of an acidifier may result in improvements in growth performance due to a decrease in gastric pH. Kil et al. (2011) reported that throughout 17 studies, 76% of the treatment groups supplemented with a dietary acidifier experienced reductions in stomach pH. This reduction in stomach pH may lead to impacts on gut microbiota and nutrient digestion. This has been validated utilizing potassium diformate which has been shown to consistently improve ADG and feed efficiency (Poeikhampha and Bunchasak, 2011; Htoo and Molares, 2012), benefit intestinal morphology (Poeikhampha and Bunchasak, 2011), and exhibit antimicrobial properties (Canibe et al., 2001) when fed to nursery pigs. The previous research utilizing potassium diformate aligns with the improvement in feed efficiency reported during the first two nursery phases in Exp. 1. However, in the present study, the improvements in feed efficiency did not continue into phase 3 or translate into differences for the overall experimental period. This contradicts a previous study (Htoo and Molares; 2012) utilizing two sources of potassium formate that observed no differences in the early nursery period (days 0 to 14), but found improvements in ADG and G:F in both the late nursery (days 15 to 35) and overall period (days 0 to 35). The ADG and feed intake for the first 14 d of the Htoo and Molares (2012) study was notably lower than in the present study. The authors theorized that this lower feed intake during the prestarter phase may have been the cause for the lack of differences at the beginning of the study, with improvements in performance seen later in the study when pigs began to consume more feed. Therefore, the response in acidifiers within the nursery period may be driven by the health and performance level of those pigs when entering the nursery or by the differences in effectiveness of the acidifier itself.

Hutchens et al. (2021) evaluated the use of 1.2% sodium diformate fed in the first two phases of the nursery (days 0 to 21). Pigs fed 1.2% sodium diformate had increased ADG and G:F. This improvement in G:F observed during the early nursery phase when using 1.2% sodium diformate aligns with the study herein, with the study by Hutchens et al. (2021) reporting a 7% improvement in G:F and the present study finding a 4% improvement in G:F. The similarity in results was expected as both trials were conducted utilizing similar research conditions, genetics, and the same sodium diformate product. However, Hutchens et al. (2021) found a tendency for an increased ADG and day 42 BW in the overall period despite only feeding sodium diformate for the first two phases. This research indicates sodium diformate can consistently impact the growth performance of pigs during the early nursery period, but further data are needed to understand how to translate those differences into the late nursery phase.

In addition to improvements in growth performance, acidifiers may have the potential to reduce the incidence of diarrhea in nursery pigs. Fecal DM analysis is often viewed as the gold standard for assessing a pig’s fecal consistency in order to understand the prevalence of diarrhea within a herd due to its objectivity (Eriksen et al., 2024). Specifically, recent work reported an increase in fecal DM as acid-binding capacity is decreased in weanling pig diets utilizing acidifiers and specialty protein ingredients (Stas et al., 2023). However, studies evaluating either fecal score, incidence of diarrhea, or fecal DM have observed no differences with the use of dietary acidifiers alone (Htoo and Molares, 2012; Grecco et al., 2018; Hutchens et al., 2021). In Exp. 1, feeding sodium diformate did not impact fecal DM on day 9, but decreased fecal DM on day 24. This response was unexpected as the previous research has shown either a positive or no response to diet acidification on fecal DM. In Exp. 1, pigs were healthy and had an adequate feed intake; therefore, fecal DM was relatively high with treatment means ranging from 24.8% to 26.9% on day 9 and 21.9% to 25.7% on day 24. Eriksen et al. (2024) identified thresholds categorizing fecal DM percentages into four fecal consistencies: firm, soft and shaped, loose diarrhea, and watery diarrhea. According to their assessment, a fecal dry matter percentage below approximately 18% would begin to fall within the loose category. Within our study, all of our treatments fell within the firm or soft and shaped categories indicative of a healthy herd. Although there was a decrease in fecal DM on day 24, it was still in a higher range compared to previous studies conducted within the same research setting for a nursery pig of that age (Batson et al., 2021; Laskoski et al., 2021). Ultimately, further research would be needed to investigate the response of sodium diformate on fecal DM during a health challenge to elicit a better understanding of its impact on the gastrointestinal tract.

Despite the previous literature outlining the positive benefits of formic acid salts on nursery pig performance, there has been little literature investigating its effects in finishing pig diets. Although finishing pigs are not limited by their capacity to produce hydrochloric acid in the stomach unlike a weanling pig, there may still be benefits to additional acidification leading to improvements in the digestibility of the diet (Liu et al., 2018). Overland et al. (2000) evaluated the use of a calcium/sodium-formate blend and potassium diformate fed to finishing pigs. Overall, potassium diformate increased ADG during the experimental period; however, the calcium/sodium-formate blend did not have an impact on growth performance. In Exp. 2, there were improvements in both ADG and ADFI which are similar to those outlined by Overland et al. (2000) when utilizing potassium diformate. The interesting comparison between these trials is the benefits reported when utilizing sodium diformate, but the lack of response when using the calcium/sodium-formate blend. This may be due to difference in the chemical structure, with the additional formate group in sodium diformate potentially providing additional acidification potential. However, this would need to be further investigated. This indicates the importance of validating each acidifier as they enter the market, even if they are thought to be similar. Most of the response to sodium diformate during the present study was found in the late finishing period (after day 60), while the improvements in ADG utilizing potassium diformate were largely found in the early grower period. Similar to the studies presented utilizing acidifiers in the nursery period, there seems to be an inconsistency in the magnitude and timing of the benefits reported when utilizing acidifiers in the finishing period. The inconsistency in response may be attributed to factors other than acidifier type including inclusion level, protein source, protein level, and health status. Further research into these factors is needed to understand the optimal inclusion of acidifiers within the finishing period.

Overland et al. (2000) hypothesized that the potential increase in protein digestibility observed when potassium diformate is supplemented in the diet may lead to an increase in lean protein deposition. In their series of experiments, they reported increased carcass lean content with potassium diformate supplementation but did not report a similar increase when utilizing a calcium/sodium-formate blend. In the present study, although pigs experienced an increase in ADG, this did not translate into differences in carcass characteristics. However, in the present study, the carcass lean percentage was higher than what was observed in the study by Overland et al. (2000) (57.3% vs. 54.3%) and may be a potential reason for the discrepancy in carcass findings between the potassium diformate and the sodium diformate product, along with the differences in the acidifiers themselves.

Although the majority of benefits in growth response from sodium diformate were seen in late finishing, there was an improvement in G:F up to 0.50% sodium diformate during the first 32 d of the study. Interestingly, this aligns with the early improvements in G:F seen in the nursery trial when including increasing levels of sodium diformate. Tung and Pettigrew (2006) identified acidifiers as being a useful tool to help pigs overcome periods of high stress and disease challenge. Therefore, the improvements in G:F may be driven by the initial introduction of an acidifier to the diet or the inclusion of an acidifier during a period of stress such as at weaning or transition to the finisher where pigs undergo transportation, introduction to a new environment, or transition to a new diet. It is important to note that there was a high health status and low levels of removals and mortalities throughout both studies. Therefore, additional research is needed to understand if sodium diformate could elicit a greater response under more challenging conditions.

In summary, these data suggest increasing sodium diformate has the potential to increase G:F in the early nursery period but did not affect performance in the late nursery. This indicates that the acidification potential of sodium diformate has the greatest benefit immediately postweaning, when the pig is the most challenged. However, the benefits postweaning did not translate into improved performance for the overall nursery period. Furthermore, feeding increasing sodium diformate increased ADG and ADFI after day 60 (approximately 82 kg) in the finishing period but carcass traits were unaffected. In both studies, improvements were seen to the highest inclusion level, 1.20% and 0.75%, respectively. Additional research is needed on target feeding sodium diformate during specific stages of the nursery and finishing period to maximize performance while being economical for producers.
